# Strengthening Global Surveillance for Antimicrobial Drug–Resistant *Neisseria gonorrhoeae* through the Enhanced Gonococcal Antimicrobial Surveillance Program

**DOI:** 10.3201/eid2313.170443

**Published:** 2017-12

**Authors:** Emily J. Weston, Teodora Wi, John Papp

**Affiliations:** Centers for Disease Control and Prevention, Atlanta, Georgia, USA (E.J. Weston, J. Papp);; World Health Organization, Geneva, Switzerland (T. Wi)

**Keywords:** *Neisseria* *gonorrhoeae*, antimicrobial, antimicrobial resistance, global health security, AMR, antibiotic, gonococcal, gonorrhea, sexually transmitted disease, sexually transmitted infections, surveillance, azithromycin, penicillin, tetracycline, quinolones, ciprofloxacin, ceftriaxone, urethral discharge, EGASP, GISP, GRASP, AGSP, Euro-GASP, GLASS, CARB, bacteria

## Abstract

Monitoring trends in antimicrobial drug–resistant *Neisseria gonorrhoeae* is a critical public health and global health security activity because the number of antimicrobial drugs available to treat gonorrhea effectively is rapidly diminishing. Current global surveillance methods for antimicrobial drug–resistant *N. gonorrhoeae* have many limitations, especially in countries with the greatest burden of disease. The Enhanced Gonococcal Antimicrobial Surveillance Program is a collaboration between the World Health Organization and the Centers for Disease Control and Prevention. The program aims to monitor trends in antimicrobial drug susceptibilities in *N. gonorrhoeae* by using standardized sampling and laboratory protocols; to improve the quality, comparability, and timeliness of gonococcal antimicrobial drug resistance data across multiple countries; and to assess resistance patterns in key populations at highest risk for antimicrobial drug–resistant gonorrhea so country-specific treatment guidelines can be informed.

Bacterial infections can cause disease ranging in severity from mild to life-threatening, and resistance to antibiotics may hamper treatment. *Neisseria gonorrhoeae* is a common, worldwide sexually transmitted infection (STI) that can lead to severe reproductive sequelae, such as pelvic inflammatory disease, infertility, ectopic pregnancy, and epididymitis. If left untreated or improperly treated, gonococcal infections can become disseminated and cause sepsis (*1**,**2*). In addition, infection may also facilitate HIV transmission (*3*). 

## Emerging Antimicrobial Drug–Resistant *N. gonorrhoeae*

*N. gonorrhoeae* has adapted to treatment through all mechanisms of antimicrobial resistance (AMR). Antimicrobial drug–resistant *N. gonorrhoeae* has outpaced novel treatment options; the third-generation cephalosporin ceftriaxone is among the most recent drugs currently being prescribed for treatment against gonorrhea. Gonococcal resistance to penicillin and tetracycline was first reported in Asia during the 1970s; resistance became widespread in multiple regions of the world during the early 1980s. High levels of resistance to quinolones (e.g., ciprofloxacin) developed by the mid-2000s; the US Centers for Disease Control and Prevention (CDC) removed these drugs from recommended treatment regimens for gonorrhea in 2007 (*4*). Public health agencies in most countries and the World Health Organization (WHO) recommend treating gonorrhea with ceftriaxone in combination with azithromycin in an attempt to slow simultaneous emergence of AMR to 2 unrelated compounds (*5*–*9*). However, recent reports suggest that this combination is becoming less effective in treating gonorrhea (*10**,**11*).

An estimated 357 million new STIs were reported among adults 15–49 years of age in 2012; 78 million of those new cases were attributed to gonorrhea (*12*). For 2012, the global incidence rate of gonorrhea among men was 24 cases/1,000 (regional range 13–41 cases/1,000); among women, the incidence rate was 19 cases/1,000 (WHO regional range 8–37 cases/1,000). Among men, estimates for the incidence rate were highest in the Western Pacific Region and second highest in the Southeast Asia Region. Among women, estimates of incidence rate were also found to be the highest in the Western Pacific Region but were documented next highest in the African Region.

Adding to global burden estimates, many countries, especially those that are resource poor, have been unable to readily implement or improve laboratory diagnostics for gonorrhea; therefore, syndromic surveillance is often used. Among male patients, WHO recommends monitoring trends in urethral discharge as an indicator of an incident gonococcal infection (*13*). However, syndromic surveillance is limited in the ability to assess the occurrence of gonorrhea because the data are difficult to interpret; other organisms, such as *Chlamydia* *trachomatis*, may cause urethritis; and some symptomatic men may not seek care. Further, inconsistencies in reporting among countries may lead to difficulties in comparing estimates (*13**,**14*). As with all communicable diseases, critical infrastructure for accurate epidemiologic practices and laboratory testing for *N*. *gonorrhoeae* should be in place for high-quality surveillance, and the ultimate goal should be to reduce the incidence of gonococcal infections. Incorporating surveillance with both epidemiologic practices and laboratory testing to replace syndromic surveillance further prepares against the threat of antimicrobial drug­–resistant gonorrhea and is imperative in enhancing global health security.

### Global Surveillance Activities for Antimicrobial Drug–Resistant *N*. *gonorrhoeae*

CDC’s Gonococcal Isolate Surveillance Program (GISP), a surveillance system established in 1986 to monitor trends of antimicrobial susceptibilities of *N. gonorrhoeae* in the United States, has been instrumental in documenting AMR patterns of gonorrhea and the spread of resistance among different drug classes. Laboratory and epidemiologic data collected through GISP are analyzed to estimate the proportion of isolates with resistance or decreased susceptibility in key populations; findings are disseminated annually (*15*). In 2014, for example, data from GISP demonstrated increases in the prevalence of reduced susceptibility to azithromycin and to cefixime. Data from GISP were used to modify and inform US sexually transmitted disease treatment guidelines (*6*).

The Gonococcal Resistance to Antimicrobial Surveillance Programme (GRASP) in the United Kingdom and the Australian Gonococcal Surveillance Program (AGSP) have also established country-specific surveillance systems to monitor trends in susceptibility to antimicrobial drugs for treatment of gonorrhea. Member states of the European Union participate in the European Gonococcal Antimicrobial Surveillance Programme (Euro-GASP). Though the methodologies of each surveillance system differ, all 3 surveillance programs analyze and disseminate susceptibility trend data similar to GISP (*16**–**18*). Data from each of these countries have also been used to inform local treatment guidelines (*7**–**9*).

On a more global scale, the WHO Gonococcal Antimicrobial Surveillance Program (GASP), which is not related to the Euro-GASP surveillance, has been collecting gonococcal antimicrobial susceptibility data since 1992. GASP is a worldwide laboratory network that is coordinated by focal points and regional coordinating centers. Each designated regional focal point, in partnership with its WHO regional office, collates susceptibility data submitted by participating countries. These susceptibility data have provided evidence to inform national, regional, and global treatment guidelines.

There are many challenges in the current GASP framework. The cumulative number of countries reporting AMR data for any antibiotic increased from 56 in 2009 to 77 in 2014. However, the number of countries reporting susceptibility data for >1 antibiotic each year shows a declining trend, from 56 countries in 2009 to 52 countries in 2014. In 2014, the WHO European Region (n = 24 countries) accounted for most reports in GASP; only 2 countries in Africa and 1 country in the Eastern Mediterranean Region provided data to GASP. The lack of reports from some regions reflects the financial and laboratory constraints of countries in resource-poor settings to implement GASP. In addition, a recent WHO survey from 108 countries showed that only 46% had conducted AMR testing for gonorrhea during the past 5 years (*13*). The AMR data for gonorrhea from GASP may be reported from differing countries from year to year, making interpretation of trends difficult (*13*).

Globally, some countries have suboptimal surveillance systems and laboratory diagnostics because of limited epidemiologic and laboratory capacity and lack of political will and funding (*4*). Very often, countries rely on syndromic management of STIs, which limits their collection of isolates for susceptibility monitoring. This results in a vicious cycle of limited laboratory capacity to conduct antimicrobial susceptibility testing (AST); thus, sample sizes for AMR surveillance are limited. As a result, some isolates obtained in GASP are not systematically collected but are convenience samples, making it difficult to generalize findings. 

In a different setting, many resource-rich organizations have used nucleic acid amplification tests (NAATs) for diagnosis of gonorrhea, which has also limited the collection of cultures for AST. NAATs in particular can detect nonviable gonococci, have high sensitivity compared to other diagnostic methods (especially for rectal and oropharyngeal specimens), can be self-collected, can detect multiple pathogens in 1 test, and can provide results rapidly (*4*). As a result, some countries in the GASP network have not collected a minimum sample size of 100 isolates of urethral discharge as recommended by WHO to confidently detect a 5% resistance, the typical cutoff point used to inform revision of treatment recommendations (*19**,**20*). These small sample sizes ultimately do not support comparison of data across countries and regions (*13*) and assessment of trends over time. There are also issues related to quality of laboratory testing. Variation in AST methods and limited capacity of laboratory methodologies for specimen collection, culture testing, AMR testing, and quality assurance procedures (*4**,**13*) can result in data that are invalid and incomparable across countries. In addition, AST results are reported as MICs, which are the lowest antimicrobial concentrations that inhibit growth of a microorganism. As MICs increase, organisms can grow at higher antimicrobial concentrations that provide an early warning or alert of impending resistance (*15*). GASP has noted that countries frequently batch test isolates, which possibly leads to delayed reporting. Such practices may compromise global preparedness for emerging resistance if any of these isolates are found to have critical MICs many months after being collected.

Finally, many countries participating in GASP do not routinely obtain demographic, behavioral, or clinical data with the isolates, so it is not possible to identify and understand epidemiologic factors or known behavioral risk factors associated with resistance. Global surveillance for gonococcal AMR should be strengthened, especially in the most disease-burdened countries, where the greatest need for AMR monitoring is essential. Because of the impending spread of resistant extended-spectrum cephalosporins, monitoring AMR is essential to inform treatment guidelines and policy, as well as interventions for gonococcal infections. As part of a collaborative effort between WHO and CDC, sentinel countries are being strategically selected to enhance the GASP program. Selected countries will link valid and comparable laboratory data to epidemiologic data to establish mechanisms for early warning of emergence of antibacterial drug resistance to inform national and global treatment guidelines and policies.

### Enhanced Gonorrhea Antimicrobial Surveillance Project Implementation

A 2013 CDC report categorized antimicrobial drug–resistant gonorrhea as an urgent threat that required immediate and aggressive action (*21*). Data in that report were primarily from GISP (*15*). The contents of the report helped spur the National Strategy to Combat Antibiotic Resistant Bacteria (CARB). A basic tenet of CARB is to improve international collaboration and capacities for AMR prevention, surveillance, control, and antibiotic research and development. To enhance this global response, CDC was encouraged to collaborate with countries to strengthen antibiotic stewardship and help ensure that laboratories around the world could identify and report resistant bacteria (*22*). Recognizing the need to establish a robust sentinel surveillance program for emerging drug-resistant *N. gonorrhoeae*, and reflecting on the known successes of GISP (as well as evidence from other country surveillance systems) and the call to enhance global health security, WHO and CDC developed the Enhanced Gonococcal Antimicrobial Surveillance Program (EGASP) in late 2015.

The primary objective of EGASP is to monitor trends in antimicrobial susceptibilities in *N. gonorrhoeae* by using standardized sampling and laboratory protocols at selected sentinel sites and reference laboratories. A second objective of EGASP is to improve the quality, comparability, and timeliness of gonococcal antimicrobial resistance data across multiple countries. Further, EGASP aims to assess resistance patterns in key populations at highest risk for antimicrobial drug–resistant gonorrhea to eventually inform treatment guidelines and other policy measures.

Prior to implementation, EGASP coordinators make an assessment site visit. Selection of countries for EGASP implementation is based on *N. gonorrhoeae* morbidity, ease of access to healthcare providers, competent laboratory services, government engagement, and a partner in the country (such as a WHO or CDC country office) that will help champion the project when technical advisors are not in the country. Although a minimum threshold of *N. gonorrhoeae* morbidity has not been established because of limited available data, countries that have been able to document a high rate of identified gonorrhea cases are prioritized for this surveillance activity.

Urethral specimens are collected from patients at selected clinics who had symptoms suggestive of gonorrhea (i.e., urethritis, dysuria); a Gram stain test is done, and a culture is sent to a participatory laboratory for processing. Before the patient leaves the clinic, antibiotics are prescribed on the basis of the results of the confirmed Gram stain. Treatment is provided on the basis of local treatment guidelines for gonococcal or nongonococcal urethritis; treatment is provided for both gonorrhea and chlamydia infections.

Bacterial identification testing is performed on any culture isolates; those confirmed to be *N. gonorrhoeae* are tested for susceptibility to specific antimicrobial drugs currently recommended to treat gonorrhea by using Etest (bioMérieux, Durham, NC, USA). Etest was selected for MIC determination in EGASP because it is comparable to agar dilution but available at a lower cost and enables the calculation of trends of drug susceptibility data over time (*4*). AST results for ceftriaxone, cefixime, and azithromycin are recorded at each EGASP surveillance site for analysis. The EGASP country may additionally select other antimicrobial agents for assessment depending on local interests. Because of the considerable quality assurance and control methods that are required with the use of Etest, laboratory personnel are required to complete a CDC training program and pass 2 annual quality assurance proficiency tests administered by CDC.

Behavioral and clinical data, such as demographics, prior antibiotic use, sexual behavior history, and treatment, are collected on a case abstraction form for each person enrolled in EGASP. Persons who have a positive *N*. *gonorrhoeae* culture are enrolled into EGASP and their isolates are submitted for AST. EGASP continuously enrolls symptomatic persons with confirmed *N. gonorrhoeae*. A coordinator is assigned at each sentinel site and is responsible for data collection (including the review of the abstraction forms to ensure that all questions have been reviewed before a person leaves the clinic); appropriate gonococcal isolate collection; confirming that all isolates are sent to the designated reference laboratory; and ensuring that all surveillance and laboratory personnel are adhering to the clinical, laboratory, and data standard operating procedures. Data from the sentinel sites and laboratories are later merged and sent to the Ministry of Public Health or equivalent, and monthly progress reports are sent to WHO and CDC for quality assurance and technical assistance review.

The first EGASP site was implemented in late 2015 in Bangkok, Thailand, where 2 sentinel sites and 2 reference laboratories were selected. In addition to WHO and CDC, partners include the Thai Ministry of Public Health (MOPH) and CDC’s Thailand Ministry of Public Health–US Centers for Disease Control Collaboration (TUC); CDC staff are from the Division of HIV/AIDS Prevention, National Center for HIV/AIDS, Viral Hepatitis, STD and TB Prevention. One sentinel surveillance site in Thailand, Bangrak Hospital, is the largest STI center in Bangkok and has several clinics on campus; currently, 2 clinics contribute specimens for EGASP. Silom Community Clinic at the Hospital for Tropical Medicine, a CDC partner that has been involved in community HIV trials, is the second sentinel surveillance site in Thailand.

EGASP Thailand currently collects specimens from all symptomatic male patients with urethritis. Both laboratories passed their first 2 external quality assessments. Their own internal quality control assessment of surveillance found several items that led to updated standard operating procedure instructions and development of a surveillance training course. Analysis of EGASP data from the first year of surveillance is currently under way; description of the cases and the antimicrobial susceptibility distribution tables will be published in a WHO/CDC report in 2017. To date, however, ≈1,100 male patients who had urethritis have been enrolled in Thailand EGASP; samples from 54% of male patients were confirmed as culture-positive for gonorrhea. AST was performed on 590 isolates; all of these also have a respective chart abstraction showing clinical and epidemiologic data.

In fall 2016, WHO and CDC completed an initial assessment EGASP visit to a Western Pacific Region country to determine if surveillance could be initiated in this region. According to current plans, EGASP surveillance will be established in 1 Western Pacific Region country by the end of calendar year 2017. The vision for EGASP is to include at least 10 sentinel-enhanced countries through all 6 WHO regions. WHO and CDC plan to implement EGASP surveillance in a country after appropriate assessments (i.e., review of *N. gonorrhoeae* morbidity, ease of access to healthcare providers, competent laboratory services, government engagement, and an in-country WHO or CDC partner) have been made. Depending on global AMR funds, the plan is to implement EGASP surveillance for the next 4–6 years. Afterward, WHO and CDC hope that participating countries will serve as examples for other countries, and the 2 agencies will share a generic EGASP protocol and standard operating procedures with interested countries. WHO and CDC plan to release annual reports of results from EGASP surveillance.

In addition, unlike GASP, where country-specific data are currently reported directly to regional focal points, the reporting of EGASP surveillance data are being implemented into WHO’s Global Antimicrobial Surveillance Systems (GLASS) to ensure sustainability and country ownership. The WHO Antimicrobial Secretariat coordinates GLASS; this division is responsible for strengthening AMR surveillance of bacteria, viruses, and fungi; *N. gonorrhoeae* is 1 of 9 priority bacterial infections that are being monitored. GLASS is being launched to support a standardized approach to the collection, analysis, and sharing of AMR data on a global level; countries will enter EGASP data into the GLASS system, and the surveillance data will be submitted directly to WHO and CDC (*23*). In addition, while it is envisioned that GASP will soon report country-specific data through GLASS, at this time, WHO plans to commence with EGASP in this system first.

## Conclusions

Consistent and systematic surveillance for antimicrobial drug–resistant gonorrhea is essential to assess if, when, and how resistance is spreading globally and to inform national and global action to control and mitigate AMR in gonorrhea. Globally, it is a critical time in which the capacity for both culture and AST need to be strengthened and implemented where absent. Building sustainable surveillance systems is the key to understanding trends; having the knowledge of global patterns and trend data puts all stakeholders in a better position to understand threat levels and to prepare action and response plans. Although EGASP is still in its infancy, its goals are the implementation of strong epidemiologic and laboratory capacities and the ability for WHO and CDC to provide technical assistance to many countries. This surveillance program is designed to enable each country to assess resistance patterns in key populations at highest risk for antimicrobial drug–resistant gonorrhea, and enables countries to use these data to inform their own treatment guidelines. In addition, EGASP contributes to the global picture for standardized surveillance, as it will assist us in understanding global and regional trends for antimicrobial drug­­­–resistant gonorrhea and will facilitate targeted response to global health threats. This international preparedness plan of implementing strong surveillance to detect resistant gonorrhea replicates other efforts currently being undertaken at CDC and WHO and serves as a lesson learned from the 2014–2015 West Africa Ebola response as a way to enhance overall global health security.

Establishing surveillance systems to monitor the emergence and spread of antimicrobial drug–resistant *N. gonorrhoeae* supports the development of evidence-based, regional treatment recommendations rather than the current approach that is based on a few systematic surveillance systems. This process would support the evaluation of treatment recommendations and, in some cases, may permit for more treatment options if antimicrobial susceptibility patterns differ globally. EGASP will build capacity in countries to conduct robust surveillance by facilitating the collection of relevant epidemiologic data associated with accurate laboratory results; this is a model that could be applied to other infectious agents. This clearly serves the global health security community as an early warning for antimicrobial drug–resistant *N. gonorrhoeae* and supports improved clinical activities.
